# IIT’s Scientific Counter-Revolution: A Neuroscientific Theory’s Physical and Metaphysical Implications

**DOI:** 10.3390/e23080942

**Published:** 2021-07-23

**Authors:** Francis Fallon, James C. Blackmon

**Affiliations:** 1 Philosophy Department, St. John’s University, New York, NY 11439, USA; 2 Department of Philosophy, San Francisco State University, San Francisco, CA 94132, USA

**Keywords:** consciousness, Integrated Information Theory (IIT), Tononi, metaphysics

## Abstract

IIT includes commitments about the very nature of physical reality, a fact both highly unusual for an empirical theory within neuroscience, and surprisingly underappreciated within the literature. These commitments are intimately tied to the theory; they are not incidental. This paper demonstrates as much by raising certain objections in a “naive” way, and then exposing how the principled IIT responses would rely upon metaphysical positions. Along the way we draw on the IIT literature for support for these interpretations, but also point to a need for elaboration and clarification. Section 1 applies the Placement Argument in a way that leads to problem involving zombies, treated in Section 2. Section 3 frames the zombie problem as an apparent dilemma, and addresses that dilemma by drawing on claims in the IIT literature concerning physical reality. Section 4 raises a related dilemma and treats it in a way that dovetails with the treatment in Section 3 of physical reality. All of this underscores not just the breadth of IIT, but the relevance of this breadth to a full consideration of IIT’s merits.

## 1. Introduction

The exact nature of IIT’s uniqueness is very subtle. True, it is a theory of consciousness that has implications for how we think about physical reality. However, to those who would discount such measures as extreme, the IIT proponent can respond with a wealth of precedent.

In the sciences, the modern tradition of radical proposals for explaining consciousness includes no less an august figure than Schrodinger [1]. Contemporary scientists such as Tegmark [2], Barrett [3], or McFadden [4] follow in these steps, joining with IIT in envisioning profound alterations to the way we understand the natural world in order to account for experience. Certainly, there is no shortage of philosophical theories that call for radically revising our beliefs. Deacon [5] argues that science as we know it is crucially incomplete, standing in need of serious augmentation. Chalmers and, more recently, Goff and Mørch [6] are among many who—following Russell [7]—argue that science only has the capacity to describe the structure of the physical world and that much of reality, including the nature of consciousness, lies necessarily hidden from the view of science as we know it. Notice though that these figures, unlike IIT’s founder and many of its main proponents, are not engaged in empirical, neuroscientific work on consciousness. This is the sense in which IIT is unique [8,9,10]. 

And yet the physical and metaphysical commitments of IIT are relatively neglected. In IIT’s own literature one can find plenty of metaphysical exposition, but it does not receive emphasis (sometimes tucked into footnotes) and is increasingly dwarfed by the body of work on technical and empirical matters. Occasional and brief treatments of this side of IIT do exist. Ref. [11] is one example. More usually (or at least so it seems) IIT is assessed in such a way that suggests that its theoretical underpinnings need to be and can be altered to salvage its empirical appeal [12,13]. Perhaps about as often, perhaps more often, the theoretical considerations are ignored or glossed cursorily for the sake of reference, and the sole focus is on technical matters such as the measurement of Φ [14] the fit (or lack thereof) between IIT’s predictions and the empirical data is the sole focus [15]. 

This paper provides background for—and then outlines an argument that leads to—an apparent dilemma for IIT. The response sketched attempts an informed speculation, drawing on IIT’s physical and metaphysical principles. A second apparent dilemma finds convergent resolution, adding both detail and credence to our speculations. These various objections are raised in a “naive” way, and then followed by an exposition of how the principled IIT responses would rely upon metaphysical positions. 

We leave to IIT’s proponents whether these speculations accurately represent the theory and, if necessary, to correct the record. We leave to the general reader whether the physical and metaphysical commitments of IIT are powerful new resources or theoretical liabilities. The reader should at least, we hope, come to see IIT as much more than an entry in a neuroscientific debate. For better or worse, it is a worldview. 

## 2. Preliminaries

We will show here how an attempt to better understand IIT brings this worldview to light. It all starts with (i) the observation that consciousness, on IIT, appears not to be intrinsic to the systems that have it and (ii) a consideration of how proponents of IIT might respond. In considering how IIT proponents might respond, we have strived to stay within (our interpretation of) the IIT framework as it stands (rather than suggesting significant alternations, for example). The aim is to examine what IIT is asking us to accept, not to assess its success. 

The observation that consciousness appears, on IIT, to be extrinsic is a reasonable interpretation of IIT’s identification of consciousness with Φ^max^, a property whose very name suggests this extrinsic nature. After all, a system’s having the maximum amount of any quantity is a condition that depends on acausal relations to other things. The biggest fish in the pond is the biggest due simply to how its size compares to that of the other fish, and it can lose this property under the simple condition that some other fish grew bigger. Being the biggest, and more generally having the most of anything, is not intrinsic; it depends on acausal comparisons to other things. Φ^max^ appears to depend on acausal comparisons to other things as well. If so, then whether a physical system is conscious is not determined entirely by how it is in and of itself. It is instead determined in part by how it compares to other things. However, then, depending on these merely acausal comparisons, one physical system may be conscious while a physical duplicate of it is not conscious (To put a finer point on it, such a notion of consciousness opens the door to zombies). Moreover, a physical system can lose or gain consciousness due not to any physical changes it undergoes but to changes in conditions elsewhere. 

Blackmon [16] argues that IIT inadvertently implies that consciousness is not intrinsic using a mereological placement argument intended to show that if consciousness is Φ^max^, then a physical system may be conscious while a physical duplicate of it is not. We will survey this argument in the next section. From there we will consider what we regard as IIT’s most likely response consistent with the IIT literature and elaborate on its ramifications. Evidently, IIT’s defense against the charge that it implies that consciousness is extrinsic draws on some as yet unappreciated and controversial metaphysical commitments to the nature of existence and causation. In the sections following our presentation of the rebuttal, we hope to show the extent to which embracing the metaphysics of IIT would be revolutionary. 

## 3. Exclusion and Intrinsicality: The Placement Argument and Zombies

### 3.1. The Placement Argument 

After presenting a version (see also [16]) of the argument that the current version of IIT inadvertently implies that consciousness is not intrinsic, we will examine a possible objection a proponent of IIT might make. First, it should be noted that when IIT uses the word “intrinsic” in describing existence, it means “from its own perspective.” E.g., if a system exists intrinsically (or alternatively “has intrinsic existence”), then it exists from its own perspective (or point of view). IIT also may use the word “intrinsic” in the more typical sense in which a thing’s intrinsic properties are those had by any physical duplicate of it [17,18]. The Placement Argument raises the possibility that consciousness for IIT is not intrinsic in the typical sense, a controversial claim in its own right, and one IIT seemingly has its own reasons to disavow, identifying consciousness as it does with causal structures with certain properties. 

The argument refers to IIT’s Exclusion Postulate:

The cause-effect structure specified by the system must be *definite*: it is specified over a *single* set of elements—neither less nor more—the one over which it is *maximally irreducible* from its intrinsic perspective (Φ^max^), thus laying maximal claim to intrinsic existence. [19]

Tononi provides the following “didactic example”:

For example, within **ABCDE** … many candidate systems could specify cause-effect structures, including **AB**, **AC**, **BC**, **ABC**, **ABCD**, **ABCDE**, and so on. Among these, the system that specifies the cause-effect structure that is maximally irreducible from its own intrinsic perspective is the set of elements **ABC**, rather than any of its subsets or supersets [19].

IIT’s Exclusion Postulate entails that no system overlapping a conscious system may itself be conscious. There must, as Tononi puts it, be a “winner”, a cause-effect structure that “excludes alternative cause-effect structures specified over overlapping elements.” [19]. 

Consider now an instance of the argument using the didactic example above: **ABCDE**, constituted of logic gates **A**, **B**, **C**, **D**, and **E**. As established by Tononi’s exposition, the complex **ABC** has Φ^max^ and thus is conscious. By the Exclusion Postulate, other constituents such as **AB** do not have Φ^max^ and so are not conscious. 

However, consider AB*, a physical duplicate of **AB** that is not a proper part of anything like **ABCDE**. AB* is connected to simple independent units that causally affect AB* in just the way that **AB** is causally affected [20]. So described, AB* changes states just as **AB** does, but unlike **AB**, AB* can have Φ^max^, and in fact it does have Φ^max^ so long as **A** and **B** do not. Let AB* overlap no complex that has more Φ than it so that AB* has Φ^max^. According to IIT, AB* is conscious, but also according to IIT, **AB** is not conscious. Consciousness, then, is not intrinsic. Depending on relations to external things, consciousness is extrinsic. 

### 3.2. Classical Zombies in IIT

Call this the Placement Argument. If the Placement Argument succeeds, controversial implications follow. For one, **AB** is the zombie of AB* [21]. Moreover, given the success of the Placement Argument, it is likely that there will be many other such zombies inhabiting the world. A zombie is conceived to be a perfect physical duplicate of a conscious being (typically a human). Such zombies are used, often by dualists, in thought experiments meant to show that consciousness does not follow directly from a complete physical description of a conscious being. Zombies are usually intended only to be conceivable, motivating, for example, the Hard Problem or the Explanatory Gap. Zombies are not intended to be possible in a world like ours, let alone actual. However, if the Placement Argument succeeds, IIT implies that zombies very likely exist in the actual world. After all, **AB** is AB*’s zombie, and it is hard to see how there could not be instances of both, along with more complex and interesting versions, in our world right now. 

Oizumi et al. [22] describe how IIT permits the existence of “zombies” of a kind (their quotes), but the “zombies” they admit are merely functional duplicates on a very broad level, not physical duplicates as we have here. The example they give is of a feedforward neural network which is weakly equivalent (input-output equivalent) to some system with positive Φ^max^. The proposition that two systems with substantially different internal architecture can be weakly equivalent and yet differ regarding consciousness (i.e., that one can be conscious while the other is a “zombie”) is not nearly as controversial as the proposition that two systems can be physically identical and yet differ regarding consciousness. The latter rejects the view that consciousness supervenes on the intrinsic physical properties of the system which is conscious; the former does not [23,24]. Thus, according to the Placement Argument, IIT admits not only the previously acknowledged functional duplicate “zombies”, but classical zombies as well.

As Mørch [25] reports, Tononi has confirmed that Φ itself is not intrinsic because Φ depends on background conditions. While this may not have been widely appreciated literature, IIT gamely accepts some kind of intrinsicality like the one we use here. Even so, it is not immediately clear why or how a change in background conditions can change whether a physical system is conscious without changing that system in some physical way. An explication of how IIT regards the implications concerning zombies (beyond the merely feedforward variety) would be welcome [26].

### 3.3. Room to Respond

The Placement Argument works from the assumption that just as **ABCDE**’s component **ABC** exists, so does **ABC**’s component **AB**. One might hold that this is not an unreasonable assumption to make, and moreover, that it is reasonable to assume that the existence of **AB** is entailed by IIT. IIT’s Composition Postulate (italics in the original) invites such an interpretation.

The system must be *structured*: subsets of the elements constituting the system, *composed* in various combinations, also have cause-effect power within the system. Thus, if a system **ABC** is constituted of elements **A**, **B**, and **C**, any subset of elements (its *power set*), including **A**, **B**, **C**; **AB**, **AC**, **BC**; as well as the entire system, **ABC**, can compose a mechanism having cause-effect power. Composition allows for elementary (first-order) elements to form distinct higher-order mechanisms, and for multiple mechanisms to form a structure. [19]

We are told that **ABC**, which exists, is constituted by elements **A**, **B**, and **C** and that subsets of these elements compose to form subsets, or mechanisms, such as **AB**, which have cause-effect power. 

Now, as we will see, there is reason to believe that, Composition Postulate notwithstanding, IIT *does* deny the actual existence of **AB**. If so, IIT rebuts the Placement Argument as stated, for if **AB** does not actually exist, then it has not been shown that Φ^max^ and consciousness are not intrinsic, nor has it been shown that AB* has a zombie in the actual world. 

This move, however, raises its own questions: Does denying the existence of **AB** entail accepting that some existing things can be composed of things (indeed, mechanisms) that *themselves do not actually exist*? Is not there a tension between the claim that such things (**AB**, for example) have cause-effect power and the claim that they do not exist? How would this tension be resolved [27]?

## 4. Examining IIT Commitments: An Apparent Dilemma and Radical Responses

### 4.1. Intrinsic Existence and Dispositions

Defining the Intrinsic Existence Postulate, Tononi [19] writes:

To account for the intrinsic existence of experience, a system constituted of elements in a state must *exist intrinsically* (be *actual*): specifically, in order to exist, it must have *cause-effect power*, as there is no point in assuming that something exists if nothing can make a difference to it, or if it cannot make a difference to anything. [28,29]

Taken literally, it states that cause-effect power is a necessary condition for the very existence of a system constituted of elements in a state.

The Intrinsic Existence Postulate appears to bear two interpretations regarding cause-effect power, the necessary condition for existence proper. The question seems to be whether a system’s cause-effect power requires the presence of other things with which that system can causally interact, or if instead that system’s cause-effect power can be merely dispositional (We will explore these options before considering, in Section 4.4. the possibility of others).

Narrow IE: A system’s existence requires its disposition to causally interact with other things and the existence of at least one other thing with which it can causally interact.

Wide IE: A system’s existence requires its disposition to causally interact with other things. [30]

Consider a system disposed to causal interaction but situated all by itself with no genuine opportunity to interact causally with other systems of elements. This lack of opportunity is, of course, contingent due to its situation in the universe, not due to any physical properties that determine how it would causally interact were there other things with which it could causally interact. This system is disposed to causal interaction, though doomed never to exercise it. Is this system’s mere disposition sufficient for intrinsic existence? 

### 4.2. An Apparent Dilemma

This question appears to raise the following dilemma for IIT. If mere disposition is insufficient for actual existence, then serious metaphysical constraints are placed on the conditions under which things may exist, as we will show. If instead a mere disposition is sufficient for intrinsic existence, then it is unclear how resorting to the Intrinsic Existence Postulate can serve as a rebuttal to the Placement Argument.

Starting with the first horn of the dilemma, we should consider physical systems that have no other systems with which to causally interact even though they are disposed to do so. Consider then our universe. Under Narrow IE, the universe does not exist and is not actual, for there exist no additional physical things that can stand in any difference-making relations to the universe. The problem here (if, that is, entailing that the universe does not actually exist is a problem) is that Narrow IE requires the existence of at least one other object, but the universe (being what it is) is all alone, having no additional object with which to interact. Having intrinsic existence, being actual, is indeed extrinsic on Narrow IE because it depends on the existence of things external to it. If Narrow IE holds, then the mere disposition to interact causally is insufficient for existence. However, this is a very serious metaphysical constraint from a theory of consciousness: The universe does not exist. 

If instead, Wide IE holds, following the second horn of the dilemma, then, assuming that the universe has at least the disposition to causally interact, our very own universe is rescued from annihilation. However, Wide IE would seem to undermine IIT’s very rebuttal to the Placement Argument. For it would appear that **AB** has cause-effect power and therefore exits after all; were certain conditions otherwise, **AB** would make a difference to other things and other things would make a difference to it. Consequently, Wide IE does not serve the purpose of denying existence to overlapping systems with less Φ, and so if **AB** exists the rebuttal would seem to fail. This specific application of the dilemma for IIT, then, is that the theory either entails that the universe does not exist, or it cannot rebut the Placement Argument by denying **AB**’s existence.

### 4.3. Recapitulation

A brief recapitulation may be helpful, before we explore how IIT might respond. The Placement Argument, if successful, seems to pose a problem for IIT. One way to describe this uses the language of intrinsicality: The Placement Argument, it seems, shows that consciousness is not intrinsic to physical properties of the system that has it (it illustrates that background or situational realities matter to Φ levels too). As noted, IIT admits that Φ is not necessarily fully intrinsic in the sense operative here, so the Placement Argument does not necessarily amount to a charge against IIT of self-contradiction. It does seem to imply the controversial proposition that two systems can be physically identical and yet differ regarding consciousness. 

To avoid this implication, IIT might want to deny the very existence of subparts (such as **AB**) of maximally irreducible cause-effect (MICE) structures (such as **ABC**). Given this denial, there simply is no **AB** to be identical in structure to AB*, and so—to put a fine point on it—no system physically identical to (the conscious) AB* that is AB*’s zombie. IIT does seem to have the resources to make this move. Putting to one side a possible tension with the Composition Postulate (let us assume that any tension there can be reconciled), the Intrinsic Existence Postulate bears two interpretations. Under the Narrow interpretation, the disposition to engage in cause-effect interactions is not sufficient for existence. Holding this line, IIT can claim that **AB** does not exist, because it is subsumed in **ABC**, which is the entity that truly exercises cause-effect power [31].

Here, though, IIT runs into an apparent dilemma, illustrated by the (rather dramatic) example concerning the fate of the universe. “Narrow Intrinsic Existence” seems to imply the non-existence of the universe (which by definition can have no other object or system with which to interact). Again, if IIT opts for “Wide Intrinsic Existence” then it must face the problems (illustrated by the zombie discussion) it would avoid by adopting Narrow IEP.

### 4.4. IIT’s Possible Responses

#### 4.4.1. Causal Power over Itself?

Before going any further, let us explore whether we have admitted a false dichotomy. As we have constructed it, the options frame the question concerning a system’s existence by reference to *other* objects or systems. As those familiar with IIT will know, the notion of a system’s causal power *over itself* is frequently invoked. If it can be shown (or plausibly argued) that the universe has (potential or actual) causal power over itself, the lack of any neighbors with which it might interact becomes moot. 

The clearest and strongest cases, on IIT, of intrinsic existence, are those where a system of elements has maximal irreducible cause-effect power over itself. We can rule this out as a characteristic of the universe: Such maximally irreducible cause-effect structures are, on IIT, necessarily conscious. Now, one might want to preserve the possibility of a conscious universe, and we do not assume that this is absurd. IIT itself, however, rules that no subset of MICE structures can itself be conscious (by the principle of exclusion). So, if the universe were a MICE structure, it would be conscious, and no subset of it—including you, the reader—could be conscious. IIT starts (both methodologically and in its ontological commitments) from the undeniability of the existence of individual consciousnesses. So, again, this offers no help out of the current predicament. 

Even if the universe does not exercise *maximal* irreducible cause-effect power over itself, perhaps it could preserve existence via non-maximal cause-effect power over itself. IIT sometimes speaks of MICE structures having “maximal existence.” This language suggests that structures that still have causal power over themselves—though not maximally—still have existence, though not in as strong a sense. So perhaps there is a category of existence between the MICE and the purely extrinsic, for systems with non-maximal causal power over themselves (See [32] for some questions related to this way of talking about existence). Is it the case that the universe lends itself to characterization as having even non-maximal causal power over itself? If we stipulate that there are elements of the universe that do not make a difference to each other, then that puts such an option under threat. However, perhaps it is a constraint upon the definition of the universe that its parts interact. Maybe there are interpretations of physics that can help IIT here (We will have occasion to revisit the relationship between IIT and physics in Section 5). At the very least, we may point out now that any such account, however feasible, remains, to our knowledge, largely unexplicated in the IIT literature. 

#### 4.4.2. The Universe as Extrinsic

If the appeal to causal power over itself fails in the case of the universe, i.e., if the universe does not exist intrinsically in this sense, then we arrive back at the fork in the road: either it has intrinsic existence in virtue of its cause-effect power with other objects or systems (but it cannot, by definition), or it has intrinsic existence because it has the disposition to do so (but this would block denying the existence of systems of like AB, thus admitting that physically identical systems can exist where only one has consciousness—letting in zombies). 

If IIT wants to avoid both these consequences—and if it is correct to say that the universe cannot be said to exist in virtue of causal power over itself, as sketched just above—it will do so only by finding some further way to argue for the existence of the universe, one that does not have any such implications. There seems to us to be one clear path (though perhaps there are others): namely, to regard the existence of the universe as *extrinsic*. 

Before we pursue this line any further, a note on the context and possible application of what follows: We have arrived at the point of speculation about the existence of the universe as extrinsic (and what that means of course awaits elaboration), by way of a series of ‘ifs’: “If the appeal to causal power over itself fails” (we will address this eventually, but again—at the very least—the successful case has not been explicitly made); if IIT wants to avoid “admitting that physically identical systems can exist where only one has consciousness,” (presumably it does, but as noted IIT has happily and explicitly admitted—albeit less controversial—zombies before), etc. Whatever the truth value of these antecedents, it is worth noting that the following discussion of the universe as existing extrinsically, accompanied by various passages from the literature that plausibly support this interpretation, might merit attention on its own. In other words, even if the reader (IIT proponent or otherwise) has departed from us in our thinking as laid out so far, the following stands in need of attention anyway. 

##### 4.4.2.1. IIT’s God’s-Eye View

Numerous passages in the IIT literature provide direct or indirect support for interpreting its assessment of the universe as having merely extrinsic existence [33]. We will fill in the sketch of what this means or may mean as we go along. 

IIT has always, and continues to, assign priority of existence in a certain way. For example, in contrasting IIT with abstract disciplines such as “classical, extensional mereology” Haun and Tononi [34] say:

…all the ‘parts’ considered here are required to exist: in phenomenal terms, they are required to exist as components of an experience; in physical terms, they are required to exist as sub-structures of a cause-effect structure. As an example, among all possible distinctions and relations [read: all possible cause-effect structures], only those having maximally irreducible cause-effect power exist in a causal, intrinsic sense, while those that are reducible do not.

As explored elsewhere [32] IIT can be compared—profitably, and arguably to its advantage—with the philosophy of Searle on issues of intrinsicality and extrinsicality.

From a God’s eye-view, from outside the world, all the features of the world would be intrinsic, including intrinsic relational features such as the feature that people in our culture regard such and such objects as screwdrivers. God could not see screwdrivers, cars, bathtubs, etc., because intrinsically speaking there are no such objects. Rather, God would see *us treating* certain objects as screwdrivers, cars, bathtubs, etc. [35].

What exists from such a God’s-eye View on IIT? Of all the cause-effect structures, only “those having maximally irreducible cause-effect power exist in a causal, intrinsic sense”. These are, ex hypothesi, conscious beings. So only conscious beings exist intrinsically. These would be visible to God. What about things like screwdrivers? Discussing just such things, Tononi [19] writes:

A way to visualize the meaning of the axioms/postulates is to apply them to an everyday object, such as a light bulb. Existence: The light bulb has cause-effect power (albeit only extrinsically), since one can affect it (screw it in) and it can have effects (produce light). Composition: It is composed of multiple parts (screw base, glass bulb, filament, wire, stem, etc.), all of which have cause-effect power alone or in combination. Information: It is what it is, meaning it has the “form” of a light bulb, thereby differing from a large number of other objects (such as a fan, a chair, a table, a shoe, and so on). Integration: It cannot be subdivided without loss into causally non-interdependent parts (if you split it in two, it will not work). Exclusion: It has borders—it is neither less (just a filament) nor more (a chandelier) than what it is.

These remarks about the lightbulb and its parts having causal power would seem to admit a more generous ontology than expected, but Tononi expressly heads off such an interpretation:

Of course, while this analogy may be illuminating, it is also potentially misleading, since a light bulb exists extrinsically (it is an extrinsic “form” in space-time), whereas an experience exists intrinsically (it is an intrinsic “form” in cause-effect space).

Our local concern lies in the status of the existence of the universe. Let us dwell on the last lines of the above quotation: Space-time (let us assume that holds the same place, so to speak, as the universe) is a realm of extrinsic forms. By contrast (getting this from the “whereas”), cause-effect space (again, where the real things are, for IIT) is the realm of intrinsic forms such as (which just are?) experiences. 

The claim here is not that there are two distinct realms in a Cartesian sense (perhaps others would want to press that interpretation), but rather that there is a hierarchy of senses in which things exist. All indications here point to a priority for intrinsic, conscious existence in cause-effect space, and a derivative status for space-time, or at least what populates it. This derivative status excludes, of course, conscious beings, if indeed this hierarchy permits us to say that conscious beings exist in space-time. Perhaps it is better to say that space exists in us?

When we sketched the apparent dilemma using the case of the universe, whatever notion of universe with which we were operating, implicitly it was not intended to mean anything as specific as intrinsic cause-effect space, and was much closer (again, implicitly) to some bland (and admittedly blank) concept of space-time. (We imagine that this was the natural reading of it, too.) Given this, and if we are correct in interpreting IIT as downgrading space-time’s existence status, then it follows that the God’s-eye view, on IIT, beholds conscious beings as existing, intrinsically, and beholds a realm—i.e., the universe, or space-time—of screwdrivers, lightbulbs, and (presuming there are no surprises about the causal dynamics among the elements of such things), planets, stars, and galaxies whose existence is (extrinsic only and so) contingent upon their nature as substructures within (intrinsic) conscious, maximally irreducible cause-effect structures (or upon their nature as superstructures, within which such MICE structures might be found). I am in the universe, but—and more properly—the universe is in me [36,37].

##### 4.4.2.2. Extrinsicality and the IIT Literature

For the sake of those who regard such an inversion as a strikingly bold move, or at least a highly unusual doctrine, to ascribe to a neuroscientific theory, it will be worth quoting at some length passages from IIT, down through the ages. Here is a quotation from back in 2008:

We are by now used to considering the universe as a vast empty space that contains enormous conglomerations of mass, charge, and energy—giant bright entities (where brightness reflects energy or mass) from planets to stars to galaxies. In this view (that is, in terms of mass, charge, or energy), each of us constitutes an extremely small, dim portion of what exists—indeed, hardly more than a speck of dust. However, if consciousness (i.e., integrated information) exists as a fundamental property, an ***equally valid*** view of the universe is this: a vast empty space that contains mostly nothing, and occasionally just specks of integrated information ([Φ])—mere dust, indeed—even there where the mass-charge–energy perspective reveals huge conglomerates. On the other hand, one small corner of the known universe contains a remarkable concentration of extremely bright entities (where brightness reflects high [Φ]), orders of magnitude brighter than anything around them. Each bright “Φ-star” is the main complex of an individual human being (and most likely, of individual animals). I argue that such Φ-centric view is ***at* *least* *as* *valid*** as that of a universe dominated by mass, charge, and energy. In fact, it may be ***more* *valid***, since to be highly conscious (to have high Φ) implies that there is something it is like to be you, whereas if you just have high mass, charge, or energy, there may be little or nothing it is like to be you. From this standpoint, it would seem that entities with high Φ ***exist in a stronger sense*** than entities of high mass. [38] (emphases added)

Fairly recently, Haun and Tononi [34] published (in this Journal) a lengthy and important paper whose main thesis was to argue that IIT has unique resources for accounting for the phenomenal feel of spatial experience. In certain places in that paper, and in apparent tension with the passage just quoted, the treatment of space seems to admit something like a presupposition of a mind-independent space-time kind of universe. Here, this emerges in the context of arguing that mere representations of the environment (IIT does *not* employ representations in attempting to explain consciousness) can never bestow intrinsic experience:

Consider representations of the environment first. One might envision that the spatial structure of the external world is the referent of spatial experience, hence topographically mapped cortical areas merely need to ‘represent’ the environment, as sampled through stimulus space, to inherit its spatial structure. However, leaving aside the nature of external space itself, it is not clear how the structure of phenomenal space, as experienced from the intrinsic perspective of our conscious mind, would be inherited from something extrinsic to it. This problem is especially obvious for spatial experiences that occur when we are disconnected from the environment, as when dreaming of the starry sky. If the experience feels spatial, it must feel so because it actually has spatial properties intrinsically, when it is dreamt, and not by inheriting spatial properties from an external environment to which the brain was exposed in the past. Of course, in the course of evolution, development, and learning, causal properties of the environment do mold the neural substrate of spatial experience (see Note 21 in Appendix B) [34]

**Note 21**: *In this sense, the cause-effect structures specified by neural grids that map visual or somatosensory input should match the causal structure of the environment itself (for example, its ‘smoothness’* [34]

As this same paper points out, and as Haun has stressed (personal communication), discussions of space and spatial experience are especially difficult because we typically conflate the experience of space with its mind-independent properties…

but at issue is what corresponds to the structure of experience here and now, not how it came about. It should also be clear that stimuli from the environment are not spatially organized in themselves: there is no extendedness, no region or location, no size, boundary or distance in a sensory stimulus unless one presupposes that space already exists intrinsically in the mind of an observer [34].

In other words, extendedness, size, etc., are properties of experience (and, again, experience is intrinsic cause-effect structures), and though we cannot but ‘project’ these onto what we think of as mind-independent space, this is a mistake.

##### 4.4.2.3. A Scientific Counter-Revolution?

We interpret this (encouraged by our understanding of Haun, personal communication [39]) as a Kantian move [40]. We have already described it as an inversion, and—as is well-known in philosophical circles at least— ant’s argument that we do not perceive space and time, but rather that they structure experience (and we mistake these experiences as perceptions of them) is known as the “Copernican turn.” It is unsurprising that an inversion of understanding about the universe would call forth cosmological references, and IIT’s own language reflects this in several places. Here is a sample:

[The reliance upon] Galileo’s stance of removing subjectivity (mind) from nature in order to describe and understand [has come] come at the cost of ignoring the central aspect of reality from the intrinsic perspective—experience itself [41].

One learns early on that science consists of objectively explaining objective properties. This notion has been a cornerstone of the scientific method since Galilei, who purposefully set aside subjective properties—the way things feel to a subject—as outside the purview of science [42].

In this view, only functions matter since they can be studied objectively by independent observers. This attitude is widespread … and seemingly justified by the Galilean notion of science as the objective investigation of objective properties. Anything beyond function has been considered inexistent…, illusory,…or irredeemably phenomenal—that is, subjective—and thereby outside the scope of science [42].

These passages, in the context we have provided for them, encourage the interpretation that IIT is seeking to overturn centuries of thought. IIT proposes a Scientific Counter-Revolution [43].

To put things this way may seem provocative, but it is worth reminding ourselves that assigning priority to experience arguably simply reflects a trivial truth. As a certain stripe of philosophers—yes, Kant, but Berkeley too, and Schopenhauer [44,45]—as well as modern thinkers such as Donald Hoffman—have been comfortable entertaining and even accepting, it may be that you do not behold an external world that influences your thoughts; your thoughts (more properly, a subset of them) constitute the external world: “You don’t see with your eye, you perceive with your mind” [46,47]. While we would encourage readers to keep an open mind about these apparent metaphysical commitments of IIT, it is worth reiterating that articles in neuroscience journals do not typically veer into such (counter-) revolutionary territory as this. Consider the following remark: “it is ironic that Descartes and others considered extendedness the defining property of matter—the res extensa—in contrast to mind—the res cogitans—which was not extended” [42]. As we read it, this is neither metaphor nor hyperbole, but a consequence of IIT: it is not that space-time has extension, but rather that the mind imposes it. 

#### 4.4.3. Resolving an Apparent Inconsistency: A Speculation

If so, IIT may well have a way to avoid the dilemma and associated problems we described above. Its success will instead depend upon empirical evidence (a hotly contested, ongoing matter) and other theoretical concerns such as self-consistency. We have in some places suggested ways in which different shades of meaning concerning intrinsicality or existence might be squared with one another within the framework of IIT, generally advocating for a charitable view of its self-consistency. Certainly, its originator, Tononi, can be described as a systematic thinker, and the IIT literature typically reflects that a definite worldview is present to mind for those who work in this new tradition. One apparent inconsistency, arising from considerations mentioned above, concerns the references to evolutionary and developmental constraints that shape the cause-effect structures. Recall:

Of course, in the course of evolution, development, and learning, causal properties of the environment do mold the neural substrate of spatial experience (see Note 21 in Appendix B) [34] (Section 4.8)

**Note 21**: *In this sense, the cause-effect structures specified by neural grids that map visual or somatosensory input should match the causal structure of the environment itself (for example, its ‘smoothness’* [34] (Appendix B)

Again, the immediate context is the point that mere representations of the environment for [48] the cause-effect structure would not explain experience’s feeling (there is no extendedness in the environment, there is no color there…). However, Haun and Tononi seem to be operating with some notion of a robust environment with causal properties of its own. Have not they committed, though, to downgrading the status of the environment to derivative?

We suspect the answer to this is something like the following: IIT’s ontology is not solipsistic. Other cause-effect structures beyond one’s mind exist and, for the mind in question, constitute its environment, broadly speaking. From this pool are stimuli that of course cannot be known in themselves, but nonetheless and by definition engage in cause-effect interaction with our minds (which are themselves cause-effect structures perfectly capable by definition of giving as good as we get) [49]. The universe is the whole system (of interacting cause-effect structures), updating itself according to the rules laid out by IIT. One can glimpse how such a system might account not only for space, as we have addressed recently above, but also for time: interactions, or updates, taking place, might be constitutive of time. This is, again, speculation—we hope both plausible and charitable—but stands in need of confirmation by a canonical IIT source. 

## 5. Back to Basics: Another Apparent Dilemma and More Radical Responses

In this section we see that another dilemma, sharing parallels with the one already discussed, leads to a reconsideration of fundamental IIT commitments. This reconsideration dovetails with the metaphysical account offered above.

### 5.1. Another Apparent Dilemma

Following [50], consider a lone electron that has no other things with which to interact either because there are no other things in existence or because they are too far away to permit causal interaction even at the speed of light. Given this condition and the laws of physics as we know them, there is no sense in which the lone electron can make a physical difference to anything else that exists because nothing else exists, or at least nothing else exists that is sufficiently close. In this sense, our solitary electron is such that “nothing can make a difference to it” and “it cannot make a difference to anything.” But does not our lone electron nevertheless still have cause-effect power? After all, *had* there been, say, a photoelectric plate moving relative to our lone electron in the right way, the electron would have caused a flash of light upon colliding with that plate. So, given only the laws of physics (no condition that there are no other things), it seems possible that our electron could make a difference to some other things and that other things could make a difference to it. 

Recall our previous two interpretations of the Intrinsic Existence Postulate. On Narrow IE, our electron does not have cause-effect power and thus does not exist. Lone electrons are impossible. Wide IE, on the other hand, permits the lone electron to have cause-effect power, and thus to exist, despite the fact that there are no other things with which they can causally interact.

Wide IE, then, would appear to be the favorable interpretation for those who find the possibility of lone electrons to be quite plausible. If IIT embraces Wide IE, then at least lone electrons can exist merely on the basis of their potential to causally interact with other things, not their actually doing so. Wide IE preserves a set of presumably common intuitions about the conditions under which electrons can and cannot exist. In this sense, it would appear to be the more conservative of the options.

However, recall that all this reasoning about interpretations of intrinsic existence is being carried out because we are working under the assumption that IIT can defend itself from the Placement Argument by claiming that while **ABC** and AB* actually exist, **AB** does not. However, if lone electrons can exist on the basis of their potential for causal interaction, why cannot **AB**? After all, even if there is some sense under IIT in which **AB** is not actual or realizing any cause-effect power in relation to **C** or other elements of **ABCDE**, it is hard to see how **AB** is also not even disposed to causally interact with other systems or elements under other conditions. Unless it is not even so disposed, **AB** must exist by IIT’s lights after all. It would also seem that, prior to accepting IIT’s commitments, we have reason to see **AB** as an existing system not simply because we can point at it and talk about it (IIT would for these reasons grant AB extrinsic existence), but also because **AB** could be circumscribed incidentally, say, by a shaft of light or an X-ray beam falling on or passing through it and only it. **AB**, then, appears to exist independently of the intentions and even the existence of observers. 

The narrow interpretation of the Intrinsic Existence Postulate by which IIT avoids the consequence that AB* has **AB** as an unconscious duplicate—a zombie—is an interpretation by which lone electrons cannot exist. Thus, the lone electrons consideration challenges IIT either (1) to accept Wide IE, the existence of both the lone electrons and **AB** (zombie duplicates), (2) to accept Narrow IE, rejecting the existence of both (contrary to presumably widely held intuitions about the plausibility of the existence of lone electrons), or (3) to explain how IIT can accept the existence of lone electrons but deny the existence of **AB**. 

### 5.2. Basic Units and Cause-Effect Power

Our treatment of IIT so far suggests that one cannot casually assume that it will accord neatly with intuitions about the physical world. Previous sections have shown that, for example, one cannot assume the mind-independent existence of space-time to be a good characterization of the universe. Perhaps assumptions about lone electrons are risky too. It will make sense to take a step back and consider IIT with respect to basic units. 

#### 5.2.1. Basic Units, Cause-Effect Power, and Intrinsicality

What would basic units be, on IIT? Presumably existence is a property that attaches to basic units. To spell it out, for a unit to be truly basic, it must exist unto itself (otherwise it would not be basic). Observer-dependent existence is insufficient. In IIT’s terms, then, basic units must exist not extrinsically, but intrinsically. So, on IIT, they must have causal power over themselves (where causal power must be maximal, at least until they become subsumed into a larger MICE complex). 

What is it for a unit both to be basic and to have causal power over itself (i.e., intrinsic existence)? Tononi and Koch [51] discuss at least a *rather* basic system:

A minimal system consisting of two interconnected neurons satisfies the criterion of intrinsic existence because, through their reciprocal interactions, the system can make a difference to itself.

By the context, we read the “minimal” here not to mean utterly basic in a physical sense but rather to act as a descriptor to indicate that the dual-neuron system is both a toy example and genuinely satisfies the conditions for causal power over itself and therefore intrinsic existence. For our purposes here, the important point is just that, as noted elsewhere in [51]. 

Note that, from the intrinsic perspective of the system, integration requires that every part of the system has both causes and effects within the rest of the system, which implies bidirectional interactions. So basic units on IIT must have two input (e.g., on/off) and two output states, which by allowing for it give itself feedback (or inform itself) allows for causal power over itself. 

#### 5.2.2. Naming Basic Units on IIT

Among themselves, IIT insiders have used the phrase “intrinsic units” to denote the most fundamental physical things in their ontology. However, it is unclear whether this term could also cover any MICE structures, rather than just the most basic building blocks. The term “atom” is originally and etymologically about things which cannot be cut into further parts, but using the term here might introduce needless confusion, for not only was it long ago commandeered by physics, but it is associated more broadly with a particular set of models that we have reason to believe might not fit IIT. “Monad” would remain too strongly associated with Leibniz’s use and moreover would stand in at least superficial tension with IIT’s requirement that a basic unit have enough structural complexity to inform itself. “Dyad” respects the latter requirement but connotes something non-basic. The simultaneous complexity and simplicity pose a problem not unlike that faced by St. Patrick, who invoked the shamrock as an analogy to explain that something could be simultaneously unitary and differentiated. A neutral label, and preferably one that is identifiably related to IIT, is needed to capture this.

Following IIT’s use of “intrinsic units”, we will use “intrinsic basic units” or “IB units” to pick out whatever these most basic elements of IIT are that are supposed to be somehow both ontologically simple and yet complex enough to encode information in the form of a bit. We include ‘basic’ to connote fairly clearly that the term covers only the most basic building blocks, excluding MICE structures and other more complex structures. IB units can be thought of as the “IITy-bits” in IIT’s ontology. (Moreover, “intrinsic basic” and “IITy-bit” share the initials “IB.”) The association from “bit” with the property of being binary is propitious, gesturing toward the requirement for these units to have input/output structure. The association with information is of course quite appropriate [52,53].

#### 5.2.3. IB Units and Electrons

Can electrons, whether lonely or among neighbors, be IB units? It is unclear. In favor is the observation that electrons can have spin up and spin down, which might count as output. Additionally, perhaps the orientation of the magnetic field could count as input. It is possible that electron self-interaction could satisfy the conditions for existence on IIT. This is highly speculative, and to our knowledge there is nothing in the IIT literature that could be construed as direct support for this position (Moreover, there is reason to doubt that electrons could be IBs, as we shall see). For now, we bracket this question. Our immediate goal is to try to follow the IIT logic with respect to IBs in an attempt to address the apparent dilemma posed at the beginning of this section.

### 5.3. Alone Again

Let us return to the apparent dilemma now, but replace the electron with an IB unit:

Consider an IB that has no other things with which to interact either because there are no other things in existence or because they are too far away to permit causal interaction even at the speed of light. Given this condition and the laws of physics as we know them, there is no sense in which the IB can make a physical difference to anything else that exists because nothing else exists, or at least nothing else exists that is sufficiently close. In this sense, our IB is such that “nothing can make a difference to it” and “it cannot make a difference to anything.”

Now, when we had been considering the lonely electron, we had recourse only to two options: (1) to accept Wide IE, the existence of both the lone electron, on the basis of its potential (albeit doomed never to be realized) to interact with (hypothetical) neighbors, which logically entails accepting **AB** (and therefore zombie duplicates), or (2) to accept Narrow IE, rejecting the existence of both (contrary to presumably widely held intuitions about the plausibility of the existence of lone electrons) [54].

There, we were speaking of electrons, and moreover in an initially naïve sense, i.e., one innocent of any ties with IIT requirements for existence. So we seemed to be left with a true dilemma. We have since refocused on what IIT speaks more directly about, which we call IB units (or IBs, for short). We have bracketed the question of whether these include electrons. Now we have principles to inform our consideration of that issue: If an electron can satisfy the conditions of IB units, then its existence is secured by that fact. If electrons cannot satisfy the conditions to be IBs, then the lonely electron does not exist.

Recall this article’s very first apparent dilemma, concerning the existence of the universe. We briefly entertained, but then had to dismiss, the possibility of a third option: Perhaps (we ventured) the dilemma could be rendered false and IIT could salvage the existence of the universe (without any zombie implications) if the universe had the right kind of causal powers. However, for reasons already explicated, that turned out to be a false hope. Not so here: IBs, by stipulation, have the right kind of causal powers to exist, even in a lonely state. So there is no dilemma of the lonely IB. Wide IE would indeed compel acceptance of undesirable zombic implications, and the other horn, Narrow IE, is still unacceptable, because it requires the existence of *another* object. However, these do not exhaust the options: the IB’s causal powers over *itself* secure its existence, which preserves something like the intuitions ruled out by the dilemma of the lonely electron. 

### 5.4. Implications

#### 5.4.1. From IB Units and the Universe…

So, we have seen that characterizing basic units as IBs has theoretical virtue. At the very least, IBs—the building blocks of reality—can exist on their own. This matches our intuitions that about the very nature of building blocks. We have until now bracketed the question of whether electrons or any other fundamental particles of our standard physics can be IBs, which would be one way in which IIT’s account of building blocks could further match intuitions and would help align IIT with physics as it is currently understood (which alone would be valuable). 

Aware that IIT explicitly ties intrinsic existence to cause-effect power in the ways that it does, van Stekelenburg and Edwards [24] note the following:

In physics an individual dynamic unit U has no *direct* influence on itself, only on non-U. The negative charge of an electron does not repel itself.

If this is so, then it hard to see how electrons can be IBs. This by itself is not damning of course, but their broader point is even more serious, because it concerns MICE systems generally. 

The objection holds that the very notion of a system’s (IB’s or otherwise) causal power over itself cannot be squared with fundamental principles of physics. According to van Stekelenburg and Edwards ([24], pp. 147–148):

At the root of the problem is the intuition, shared by Descartes (1641), Leibniz (1714), James (1890), and other metaphysicians that there is no such thing as a point of view from A+B, if A and B are discrete dynamic units—which they must be if there are causal relations between them. This intuition relates to the physicist’s law of locality. Detailed formulation of the law has changed as physics has evolved. However, the primary requirement, more fundamental even than limiting propagation of causation to the speed of light, is that every causal relation (such as a billiard ball collision) has a *specific address* in a network of relations evolving in space-time.

IIT introduces the notion of a system’s informing itself, but, to van Stekelenburg and Edwards, this cannot be properly specified. To claim as much would be to take “an illegitimate self-reference step” that is “rather like the liar paradox.” Take a system AB, made up of A and B, purported to have cause-effect power over itself. As noted, any causal relation has to have a specific address in space-time. Relations of parts (A and B) to each other do not pose a problem, as they can be specified (given addresses). Van Stekelenburg and Edwards claim that A has an identity relation to itself, and not, contra IIT, a causal one. However, Tononi et al. [51] “seem to suggest that this is A+B acting on A+B. However, if A did not act on A it can only have acted on B” (p. 147). 

It is important to note that we are not endorsing van Stekelenburg and Edwards’ arguments [55,56]. We raise them deliberately, though, because they do seem to warrant a response. As of writing, there has not been (to our knowledge) a response from IIT in the literature. It may be, however, that we can provide a sketch of a response. 

IBs have to have parts in some sense, as we have covered, so the argument would seem to apply to them (as well as to larger-scale systems). Positing infinitely divisible parts to reality (denying IBs) just seems to compound the matter (and likely invites other problems). Perhaps we could deny existence to the parts of IBs: they are made out of non-existent things (just like, in the didactic example, ABC exists but AB does not). This has the virtue of holding consistent with the rest of the account and providing some answer to the objection. 

According to this way of thinking, things really do bottom out in MICE structures, and reality builds out from there. If indeed this violates contemporary physics, then perhaps IIT would claim that the problem lies with contemporary physics. Certainly, van Stekelenburg and Edwards seem to be offering arguments set against a backdrop of space-time (the crux of their account concerns space-time addresses) that we have seen does not sit well with IIT. IIT might take the line that van Stekelenburg and Edwards have given—albeit unintentionally—an account of a precise way in which the reductionist and third-person physics of Galileo through our own time fails to capture things *at our point of view*. If this is right, then, as foreshadowed at the beginning of this section, another apparent dilemma (that of the lonely electron) has dovetailed (via a consideration of basic units on IIT, and a sketch of a defense of these and MICE structures more generally against objections from van Stekelenburg and Edwards) with the metaphysical account offered in response to the first of the article’s dilemmas (concerning the universe): In short, we are led again by IIT to Counter-Revolution. 

#### 5.4.2. To Neuroscience and Everything

As ever, lurking in the background is the apparent paradox of speaking in one breath of **AB**’s causal powers and its non-existence. It would seem that the Composition Postulate—which speaks of the subsets of a system, and of them having causal power—and the Intrinsic Existence Postulate—which would deny existence to many of those subsets—contradict. For this not to be the case, perhaps it must be that the former is indeed, as floated earlier, expressed in a “wider” idiom. In this case, the confusion arises from the IIT literature sometimes meaning “extrinsic existence” when it says “existence,” and other times meaning “intrinsic existence” when it uses the term “existence.” We close with some further speculations about the meaning of “existence” on IIT. 

We have seen that, without imperiling the universe, IIT can defend against the Placement Argument by denying the existence of AB*’s zombie twin **AB**, thus escaping the charge that IIT’s consciousness is not intrinsic. **AB** is subsumed by the existing **ABC** into nonexistence. We have also seen how this ties in with other ontological commitments. Generalizing these, and applying them to neuroscience, raises further questions.

Consider the physical substrate of consciousness (PSC) in some human brain and all its proper parts. Just as **AB** is held not to exist for the reason that it is subsumed by **ABC**, it would seem that all the proper parts of the human brain’s PSC do not exist (If, for instance, the left hemisphere, or the thalamus, or some subregion of either is subsumed by the PSC, then at this moment, the left hemisphere, the thalamus, or that subregion does not exist). Perhaps the uses of the term ‘exists’ in IIT do not always conform to the standard view on which the term is used as a quantifier, not a predicate?

Consider the difference between **AB** or your left hemisphere on one hand and the Tooth Fairy or the spherical cube on the other. The latter fail to exist in a way the former do not, for we (at least in our adulthood) do not have experiences that tempt us to assert the existence of the Tooth Fairy or the spherical cube. We do, however, have experiences that lead us to assert that mechanisms such as **AB** and brain regions such as the left hemisphere exist. This holds not simply because we can take ourselves to be looking at such things and making discoveries about their properties and relations, but also because we take them to compose other existing things such as **ABC** or the whole brain, and to play causal and explanatory roles regarding the natures of **ABC** or the whole brain. Not so with the Tooth Fairy or the spherical cube; we merely fabricate. The question then is whether **AB** and your left hemisphere truly fail to exist in the way the Tooth Fairy and the spherical cube do not exist, or instead, whether IIT’s denial of their existence amounts to something less existential. 

Perhaps instead at least some uses of the term ‘exists’ should be taken as predicative, not quantitative. Such attributions of existence might be better understood as shorthand for being more causally relevant, or for having sufficient causal power or explanatory power. Here the reasoning again converges with earlier speculation (see Section 4.4.2.1): Perhaps IIT presupposes a kind of ontological hierarchy that could accommodate everything. Within this hierarchy, **AB** and your left hemisphere would enjoy a higher status than do the Tooth Fairy and the spherical cube. If so, the former may exist in the standard quantitative sense (being the value of a variable) but fail to have the kind of causal or explanatory status IIT finds things like **ABC** and your PSC to have. 

## 6. Conclusions

The Placement Argument leads to an apparent dilemma for IIT, where it seems it must choose between the non-existence of the universe, or the existence of classical zombies. We saw that IIT can avoid the zombie horn of the dilemma by conceiving of the universe as existing extrinsically only (rather than intrinsically, or from its own point of view). Following this line brings us face to face with some of IIT’s more striking proposals about metaphysics and physics (all the more striking since they come from an empirical theory of consciousness). On one reading—and a very plausible one, given the evidence from the literature—IIT means to overturn a worldview standard since at least the Scientific Revolution. These commitments of IIT can be brought to bear on another apparent dilemma, which seems to saddle IIT with a choice between accepting zombies or ruling out the possibility of a lone electron. To assess this problem requires delving into IIT’s principles as they apply to basic building blocks, which we dub “IBs” (for “intrinsic basic units”). When we reframe things with respect to these, the second dilemma dissolves, as a third option emerges: the lonely IB’s existence is ensured by IIT’s principles and preserves some of the intuitions excluded by the original dilemma. This in turn raises questions about the fit of IIT with contemporary physics, which again leads to a consideration of the radical implications of IIT, as well as speculations about the precise meaning of “existence” for IIT. 

The Introduction to this paper discussed how the literature tends not to reflect an appreciation of the physical and metaphysical commitments of IIT. Since then, we have raised two apparent problems for IIT and shown how they can be addressed by directing our focus to just such commitments. At once, though, this highlights both the unique and the Counter-Revolutionary nature of IIT: though a player in the debate within neuroscience over consciousness, it requires profound revision, or at the very least reframing, of how we understand the nature of physical reality. It seems unavoidable: for the discussion about IIT to unfold fully demands an explicit recognition that IIT is a theory of everything.

## Data Availability

Not applicable.
